# Surgery-associated acquired hemophilia A: a report of 2 cases and review of literature

**DOI:** 10.1186/s12893-020-00872-y

**Published:** 2020-09-23

**Authors:** Umar Zeb Khan, Xiangwu Yang, Matiullah Masroor, Abdul Aziz, Hui Yi, Hai Liu

**Affiliations:** 1 Department of General Surgery, The Third Xiangya Hospital of Central South University, 138 Tongzipo Rd, Changsha, 410013 China; 2 Department of General Surgery, The Second Xiangya Hospital of Central South University, 139 Renmin Middle Rd, Changsha, 410011 China; 3 Molecular Biology Research Center & Center for Medical Genetics, School of Life Sciences, Central South University, Changsha, 410078 China

**Keywords:** Acquired hemophilia A, Acquired hemophilia surgeries, Factor VIII inhibitors, SAHA

## Abstract

**Background:**

Acquired Hemophilia A (AHA) is a rare bleeding diathesis in patients with no previous personal or family bleeding history. The diagnosis of this disease often delays due to unfamiliarity of physicians with it, which leads to its high mortality rate.

**Case presentation:**

Two cases (one 12 years old female and another 18 years old male) were admitted for right upper abdominal mass and right upper abdominal pain respectively at different times. Pre-operative diagnosis of both cases was congenital choledochal cyst. They suffered continuous gastrointestinal bleeding (hematemesis and melena) with reduced hemoglobin to 54 g/L and 60 g/L after Roux-en-Y anastomosis respectively. To investigate the exact bleeding site, Digital subtraction angiography (DSA) of case 1 showed contrast overflow at small branch of proper hepatic artery but had unremarkable result for case 2, whereas gastroscopy of both cases showed unremarkable results. Multiple surgeries were also performed for hemostatic purpose but each time no active bleeding site was found. Finally, hematologists consultation was mandated in both cases and they were diagnosed as acquired haemophilia A. However, unfortunately case 1 patient could not survive because of sever hemorrhage and infection while Case 2 of 18 years old male survived after proper haemophilia treatment catalog.

**Conclusion:**

Awareness about surgery associated acquired haemophilia A (SAHA) can facilitate quick diagnosis and lifesaving management because the mortality rate in SAHA is high due to lake of knowledge or late recognition of the disease. Bleeding always occurs at surgical sites and it can occur immediately within few hours after surgery in some cases. Hemorrhage may be severe or even life threatening and it presents a special challenge for diagnosis and treatment in a patient who has just undergone a surgical procedure. The treatment strategies for AHA include resumption of hemostasis with either recombinant porcine factor VIII (rpFVIII) or bypassing agents and immunosuppressive therapy to suppress the production of the factor VIII inhibitor.

## Background

Acquired hemophilia A (AHA) is an ultra-rare bleeding disorder, about 1.48 incidence per million occurs per year, characterized by reduction of factor VIII (FVIII) activity due to the development of autoantibodies against FVIII [1–3]. These autoantibodies breakdown and neutralize the hemostatic function of factor VIII which leads to AHA in patients having no previous personal or family bleeding history [4–6]. The bleeding in AHA is mucocutaneous*,* intramuscular, postpartum or surgical site with high mortality rate [7, 8]. The mortality potential of AHA is due to acute and recurrent bleeding episodes, which needs quick diagnosis, cessation of bleeding and suppression of the acquired factor VIII inhibitors [9]. However, mostly physicians are not familiar with AHA or recognize it belatedly, while majority of patients suffer from continuous bleeding, either spontaneous or post-trauma/procedure which causes deaths due to hemorrhage [10, 11]. AHA can occur abruptly in the postoperative period which strongly suggests that surgery was the trigger of acquired hemophilia A. The diagnosis often confirmed by a prolonged activated partial thromboplastin time (aPTT) and a low level of FVIII activity with evidence of the presence of FVIII inhibitor reported in Bethesda units (BU) [12, 13]. While in the current study, we reviewed the literature and investigated two cases of surgery-associated AHA, one in 12 years old (female) and other in 18 years old (male) with spontaneous, unstoppable gastrointestinal bleeding after surgery for choledochal cyst. It was confirmed as SAHA bleeding disorder on the basis of aPTT, FVIII activity and level of factor VIII inhibitor following surgical operation.

## Case presentation

### Case 1

12 years old, 33 kg girl was admitted to Kunming General Hospital on 3rd December for recurrent upper right abdominal pain. On physical examination, it showed upper right abdominal mass with tenderness but without any signs of jaundice. Ultrasound and CT scan showed common bile duct cyst of 12.6 × 9.1 × 15 cm. So, the diagnosis on admission was congenital choledochal cyst. Preoperative blood test showed a slightly elevated ALT, AST and bilirubin, but coagulation function test was normal with PT 11.7 s (Normal 10–15 s), INR 1.02 s (normal 0.8–1.5 s) and aPTT 45 s (normal 24–48 s). There was no family history of hemophilia, no bleeding history either. After the resection of common bile duct cyst and gallbladder, Roux-en-Y anastomosis of Hepatic duct to jejunum was successfully performed without any blood transfusion needed on 7th Dec. Cephalosporin, proton pump inhibitor and intravenous nutrition were administrated after surgery, gastrointestinal function was recovered and the liquid food was permitted on the third post-operative day (POD). But she vomited a lot of coffee color liquid and presented with melena on seventh POD. To stop bleeding, icy-saline with norepinephrine was given by a gastric tube, the hemostatics, and acid inhibitor were included in the everyday treatment catalog for 3 days, but there was no remission of gastrointestinal bleeding and hemoglobin reduced to 54 g/L despite of massive transfusion. To clarify the bleeding site, a gastroscopy and DSA were performed on tenth POD. The gastroscopy showed there was blood and blood clots in the distal small bowel, but no ulcer or bleeding was found at stomach or duodenum. DSA showed contrast agent overflowing at a small branch of proper hepatic artery. So, the presumed bleeding site was at hepatico-jejunal anastomosis and the second explorative laparotomy was undergone to stop the bleeding on the same day. The jejunal loop was found enlarged by blood clots during surgery, after removal of the clots, there was oozing but no pulsatile bleeding was found from the hepatico-jejunal anastomosis, and therefore both hepatico-jejunal and jejuno-jejunal anastomosis were redone. There was no sign of bleeding in the first 3 days after second surgery. However, on the fourth POD an intractable bleeding occurred at her abdominal incision and she got a high fever of 39.5 °C at the same time. Her general condition got worsen with a distended abdomen, and massive seroperitoneum on ultrasonography and a diagnostic abdominocentesis got bloody fluid, so the third laparotomy was done to stop the intra-abdominal bleeding. At the operation dehiscence of wound was found and peritoneal cavity was full of foul-smelling blood clots, but no active bleeding site was found. Abdominal cavity was drained with drain tube after thoroughly abdominal lavage with warm normal saline. But sadly, an overwhelming hemorrhage from wound and abdominal drain tube occurred once again on the third POD of the third operation, so the fourth laparotomy was performed on 26th Dec. About 2000 ml blood and blood clots were evacuated and a more severe peritonitis was found, but no active bleeding site identified this time too. This led us to suspect a coagulation anomaly and the hematologist was consulted. Detailed coagulation workup showed aPTT of 78.1 s, FVIII activity 5% (normal 70–150%) FIX activity 67% (normal 70–120%), so the diagnosis was surgery associated acquired hemophilia A. FVIII and blood transfusion was given immediately but no bypassing agents or immunosuppressive therapy given. Unfortunately, she suffered more severe hemorrhage and with an even worse coagulation function of aPTT 150.9 s, FVIII activity 3% thereafter, and died of severe hemorrhage and infection on fifth POD of the fourth surgery. She suffered a lot going through 4 surgeries and received 12,000 ml of blood transfusion in total.

### Case 2

18 years old, 54 kg male was admitted to the Third Xiangya Hospital for the upper right abdominal pain and fever on 12th October. Ultrasonography and CT scan showed large common bile duct cyst involving left and right hepatic bile ducts. Blood test showed a normal coagulation function with APTT of 45 s, liver enzymes and bilirubin were slightly elevated. There was no bleeding history, no family history of hemophilia either. Conservative treatment included antispasmodic agents, antibiotics, and choleretic medicine, but conservative treatment was ineffective and his symptoms got even severe and with a very high temperature of 39 °C, and his white blood cells count elevated to 20 × 10^9^/L. So, he underwent resection of choledochal cyst and gallbladder followed by Roux-en-Y anastomosis on 24th Oct. The operation went well and finished at 12 AM without any blood transfusion. However, coffee like liquid came out of gastric tube at 4 AM, on first POD, and Hematemesis and hematochezia followed afterwards. The hemorrhage was unstoppable with conservative management, and the hemoglobin reduced to 60 g/L despite of prompt transfusion. The coagulation function test showed aPTT of 47 s at the moment. So, a technique fault related bleeding was suspected and an emergency explorative laparotomy was done at 1 PM, on second POD. No blood was found in abdominal cavity, but the jejunal loop was full of blood clots. After removal of the blood clots, an oozing was found at jejuno-jejunal anastomosis and it was controlled by sutures. The bleeding seems to be stopped for 2 days after the second operation but unfortunately, gastric tube and T tube inside hepatic bile duct drained bloody liquid on the third POD of second laparotomy, and it could not be controlled by conservative treatment. Bleeding even came out of abdominal drain tube on eighth POD of second surgery, so an emergency gastroscopy and DSA were performed, but neither of them showed any remarkable results. The third explorative laparotomy was done on the same day, and the jejunal loop was found enlarged by massive blood clots inside it leaded to the rupture of jejuno-jejunal anastomosis. Both hepatico-jejunual and jejuno-jejunal anastomosis were dismantled and redone this time. A multidisciplinary consultation meeting was held immediately after the third operation, and aPTT, FVIII, FIX activity and FVIII inhibitors were checked. The results showed aPTT of 105.1 s, FVIII activity 11.2%, FIX activity 119.2%, the titer of factor VIII inhibitor level was 16 Bethesda unit (BU)/ml (normal, ≤0BU), so it was concluded that the reason of the intractable bleeding was SAHA. From 5th Nov. to 26th Nov., the treatment catalog included: (1) hemostatic agents: activated prothrombin complex concentrates (APCC) 2100iu per day (total 72 ampoules, 300iu/ampoule), fresh frozen plasma and blood plasma cryoprecipitate (total 13,000 ml), FVIII 2000 ~ 6000iu per day (total 482 ampoules, 200iu/ampoule); (2) eradication of inhibitor: intravenous immunoglobulin 2.5 g ~ 5 g per day (total 46 ampoules, 2.5 g/ampoule), methylprednisolone 80 mg per day for 7 days, plasmapheresis 4 times and the total concentrated red blood cell transfusion was 10,600 ml during this period but bleeding still could not be controlled effectively. Hemothorax, empyema, atelectasis, abdominal infection, intestinal fistula, and liver dysfunction occurred in succession. The coagulation function test results during these days are given in Table 1. On 26th Nov., FVIII was replaced by recombinant factor VIIα (rFVIIα) commenced with the dosage of 2 ~ 16 mcg per day for 10 days, the first day was 16 mcg, the second was 12 mcg, and the total dosage was 35 ampoules (1mcg /ampoule). Bleeding stopped promptly after the use of rFVIIα, but it took longer for coagulation function to return to normal. Coagulation function was abnormal with aPTT of 76 s, FVIII activity 16.8% on 12th Dec., and it returned to normal with aPTT of 40.1 s, and FVIII activity 95.5% on 19th Dec. He was discharged from hospital after 2 months, but sadly, he died of malnutrition 3 years later.

**Table 1:** Coagulation profile of Case 2 from 5th November to 26th

Date	INR	APPT (in seconds)	FVIII (in %)	FXI (in %)
November 6th	1.07	77.5	11.2	119.2
November 7th	1.01	74.1	2.7	118.2
November 8th	1.07	82.2	5.9	111.6
November 9th	1.21	74.8	6.5	95.4
November 10th	1.12	85.7	6.7	113.7
November 11th	1.1	95.7	3.8	118.1
November 12th	1.2	84.4	4	117
November 13th	1.1	68.6	16.1	114.8
November 14th	1.01	62.9	13.5	111.6
November 15th	1.06	69.4	15.3	100.7
November 17th	1.11	80.6	16.6	114.8
November 19th	1.29	63.1	35.2	60.3
November 21st	1.09	84.7	13.4	112.2
November 22nd	1.09	82.6	21.3	116
November 23rd	NA	NA	3.2	72.9
November 24th	NA	NA	5	53.5
November 25th	NA	NA	4.9	53
November 26th	1.18	150	5.8	48.3

*INR* International normalized ratio

## Discussion

The exact route of causing SAHA due to induced FVIII antibodies during surgeries is not clearly defined yet. It could be related to trauma and tissue injury which expose autoantigen to immune dysregulation accompanying surgery, or possibly to a reaction to anesthetic agents or other drugs. Surgery-associated AHA is an underdiagnosed severe bleeding disorder with a high morbidity and mortality. It can happen in a few hours to several days after surgical operation, so it is apt to be overlooked or misdiagnosed as technique fault, leading to unnecessary surgical intervention. Awareness and quick diagnosis is especially important for the proper management. General accepted algorithm for AHA diagnosis includes: measurement of coagulation function, FVIII activity and the Bethesda assay for the assessment of the FVIII inhibitor level. An isolated prolonged aPTT in a bleeding, previously healthy, and not anticoagulated patient strongly indicates AHA. The two AHA’s cases reported in this study were confirmed through aPTT, FVIII activity, FIX activity, factor VIII inhibitor and no family and previous personal bleeding history. Beside these two reported cases, 23 other cases of AHA from 1978 to 2020 in 21 articles are analyzed in Table 2. The present data and reported cases show that SAHA could happen after both minor and major surgery such as Mohs surgery and pancreaticoduodenectomy respectively. Out of 25 cases (including the two cases of current study), 12 underwent digestive tract surgeries out of which 9 underwent hepatopancreatobiliary surgery. So, the probability that certain surgical procedures, such as digestive tract surgeries, especially hepatopancreatobiliary surgery, may carry a higher risk for inhibitor formation. The median age of the patients was 65 years (range 25–86 years), the median time from surgery to bleeding was 2 days (range from 0 to 8 days), the median FVIII activity was less than 3% (range 1–15%), and the median inhibitor titer at the point of diagnosis was 9BU (range 2-117BU). Out of 25 patients, 5 died from bleeding or bleeding-related complications while 16 patients underwent one or more reoperations for the purpose of hemostasis or evacuation of hematoma. However, in analyzed cases, complete remission of plasma inhibitor was achieved in 20 of 25 cases in normal range of days (15–230 days), with median 83.5 days, while 1 case persisted with inhibitor of 6.5BU even 8 years after surgery.

**Table 2:** Reported cases of surgery-associated acquired hemophilia A (SAHA)

Case	Age/gender	Surgical operation	Time from surgery to bleeding	FVIII Inhibitor titer	Hemostatic treatment	Anti-inhibitor treatment	Time to disappearance of inhibitor	Outcome	Ref
1	27/F	Episiotomy	6 days	19BU	FIX	Cyclo	–	Death	[14]
2	77/M	Retinal surgery	4 days	NS	FVIII, Cryo	Steroids,Cyclo	NS	Remission	[15]
3	25/F	Cholecystectomy	1 day	4UB	APCC	IVIG	NS	Reop, Remission	[16]
4	47/M	Bowel resection	4 days	3.3UB	FVIII,Cryop	Steroids, IVIG, PLME	40 days	2Reop, Remission	[17]
5	70/F	Cholecystectomy	1 day	5BU	FVIII	Steroids, 6-MP	2 months	Reop, Death	[17]
6	74/F	Coronary artery bypass	7 days	16BU	FVIII, APCC	Steroids, Cyclo	–	6.5BU 8 years later	[17]
7	73/M	Prostate surgery	2 days	42BU	–	IVIG	NS	Death	[18]
8	75/M	Hernia repair	4 days	10.4BU	rFVIIa	Steroids, Aza, cyclo	7 months	Reop, Remission	[19]
9	79/F	Cataract surgery	0 day	61BU	FVIII	Steroids, IVIG	NS	Reop, Remission	[20]
10	53/M	Lumbar surgery	8 days	NS	rFVIIa	Steroids	8 weeks	Remission	[21]
11	62/M	Liver transplant	2 days	6BU	FVIII, PLME	Steroids	2 months	Reop, Remission	[22]
12	57/M	Brain surgery	7 days	2BU	FVIII	Steroids, cyclosporin	8 weeks	Reop, Remission	[23]
13	82/M	Eyelid surgery	0 day	NS	rFVIIa	Steroids	NS	Reop, Remission	[24]
14	59/M	Lumbar surgery	0 day	8BU	FVIII, Cryop,	Cyclo, Steroids	15 days	Remission	[25]
15	57/F	Pancreaticojejunostomy	2 days	117BU	FVIII, rFVIIa	Steroids	100 days	Reop, Death	[26]
16	71/M	Pancreaticojejunostomy	5 days	8BU	FVIII	Steroids, rituximab	124 days	Remission	[27]
17	86/F	Mohs surgery	0 day	NS	FVIII	Steroids	NS	Reop, Remission	[28]
18	63/M	PEG	6 days	10 BU	–	Steroids	NS	Remission	[29]
19	65/M	Lysis of adhesion	1 day	5.5BU	rFVIIa	Steroids	NS	Reop,Remission	[30]
20	79/M	Pancreaticodudenectomy	3 days	18.7BU	APCC	Steroids, Cyclo	67 days	Remission	[31]
21	55/M	Cardiac surgery	0 day	66 BU	FVIII, rFVIIa,	Steroids, Cyclo	3 months	Reop, Remission	[32]
22	62/F	choledochal cyst resection	6 days	2BU	rFVIIa, APCC	IVIG, rituximab, cyclosporin	18 days	Reop, Remission	[33]
23	84/M	Distal gastrectomy	2 days	7BU	rFVIIa,	Steroids	230 days	Reop, Remission	[34]

*NS* not stated, *BU* Bethesda units, *rFVIIa* recombinant activated factor VII, *IVIG* intravenous immunoglobulin, *APCCs* activated prothrombin complex concentrates, *PEG* percutaneous endoscopic gastrostomy, *Cyclo* cyclophosphamide, *Cryop* Cryoprecipitate, *PLME* plasma exchange, *6-MP* 6-mercaptopurine, *Aza* azathioprine

An isolated prolonged aPTT with mixing studies showing poor aPTT correction after incubation of patient plasma about 1 to 2 h with an equal volume of normal plasma in a surgery associated bleeding, healthy in the past, and not anticoagulated patient strongly demonstrates SAHA. However, FVIII activity and the Bethesda assay are necessary for diagnosis and confirmation of AHA which shows the existence of FVIII neutralizing antibodies and titrates their quantity in Bethesda units [BU] [12, 35]. Awareness of all specialists especially surgeons for this unexpected life-threatening bleeding episode is crucial.

AHA treatment requires a two-pronged approach, including the control of bleeding and elimination of inhibitor [8, 9]. First-line treatment in actively bleeding patient is recombinant porcine factor VIII (rpFVIII) [36]. Before the development of rpFVIII bypassing agents were considered the first line. The two common licensed treatment products are activated prothrombin complex concentrates (APCC, FEIBA) and rFVIIa [37, 38]. Whereas, DDAVP (1-deamino-8-D-arginine vasopressin) or FVIII concentrates can also be use in cases of low titer inhibitors. Concurrent with bleeding management, corticosteroid-based therapy with immunomodulation should be initiated in order to eradicate the FVIII autoantibodies and restore normal hemostasis [39].

### Haemostatic agents

#### Recombinant porcine factor VIII (rpFVIII)

Recombinant factor VIII (obizur) is a recently developed drug which is recommended as a first line treatment for actively bleeding patients with acquired hemophilia A in June 2018 by NHS England whereas before the development of this drug, bypassing agents (APCC and rFVIIa) were considered first line of treatment for the given disease. It acts as a co factor for factor IXa which in combination with calcium and phospholipid convert factor X to factor Xa and finally achieving hemostasis after multiple steps. Obizur has relative homology to human FVIII to achieve hemostasis in humans whilst it has less thrombotic tendencies compare to rFVIIa and is more appropriate for the treatment of patients with cardiovascular diseases. Its risk of cross reactivity after administration to patient is also comparatively less (5–10%). The standard initial dose of treatment is 200u/kg but patients have also been treated with low initial doses 50-100u/kg. The low doses study suggests that patient without cross reacting antibodies to pFVIII with active bleeding can achieve factor VIII level close to 1.0 iu/ml with 50–100 u/kg dose of rpFVIII [36]. For the appropriate dosage adjustment, the measuring of degree of cross reactivity of anti-human FVIII antibodies to rpFVIII would be a better solution for physician [40].

#### Recombinant factor VIIa (rFVIIa)

Recombinant factor VIIa (bypassing agent) can directly activate factor X present on platelets surface which have already been activated at the injury site bypassing other factors like factor VIII and IX. Recombinant factor VIIa can overcome the inhibitory antibodies function (antibodies produce against FVIII) present in AHA patients. The treatment protocol for rFVIIa is 90 mcg/kg every 2 hourly until bleeding is under control then followed by less frequent doses to avoid recurrence [38]. Recombinant factor VIIa efficacy is 100% while used as a first line treatment and about 75% in cases used in late stage of the disease [41]. The use of recombinant factor VIIa is linked with thrombotic tendencies. 7% patients treated with bypassing agents shows a thrombotic incident according to a review but it is crystal clear that life endangering bleeding outweighs the danger of clotting in most cases [42].

#### APCC

Activated prothrombin complex concentrates is factors containing concentrates derived from plasma and is administered to the patient with the doses of 50-100 U/Kg twice or three time a day with the maximum of 200 U/Kg/day till the bleeding is under control followed by further reduce doses to avoid recurrence [9]. Its efficacy is 86% in patients with AHA with the doses of 75 U/Kg two to three times a day with median of 9 doses for controlling severe bleeding according to 34 patients retrospective cohort study [37].

#### DDAVP

Sometimes the bleeding episodes are minor with lower titer inhibitors (< 3 BU), in such context DDAVP may be useful at a dose of 0.3 mcg/kg intravenously. But mostly, DDAVP treatment alone is insufficient in patients with AHA; therefore, DDAVP should be use with more effective drugs [43].

#### FVIII

Bleeding control can be achieved with the high doses of FVIII in case if no bypassing agents are available and the titer of the Factor VIII inhibitor is very low. Because the treatment response of this therapy is unpredictable therefore the use of other agents to stop bleeding should not be delayed by the use of factor VIII. Factor can be much effective when used in combination with other treatment regime e.g. immunoadsorption for removal of inhibitors temporarily [44, 45].

### Eradication of inhibitors

#### Corticosteroids

The two most common immunosuppressive agent regimens include corticosteroids alone or corticosteroids in combination with cyclophosphamide or azathioprine [2, 4]. The aim of immunosuppression is to eradicate the inhibitors rapidly which decrease the risk of patient’s bleeding. There are also some other treatment options which includes either steroids only or different combinations of high-dose immunoglobulin, rituximab, cyclosporine A and immunoadsorption [38]. Prednisolone at a dose of 1 mg/kg alone or its combination with cyclophosphamide (1–2 mg/kg) is recommended regimen for initiation of immunosuppression. The median time of corticosteroid treatment for remission of inhibitors is about 5 weeks [9].

#### Intravenous immunoglobulin (IVIG)

Infusion of intravenous IVIG has also been used but there is no verified reference to support IVIG as a single agent to deal with bleeding. A total dosage of 2 g/kg over 2 ~ 5 days resulted a response rate of 30% and patients whose initial Bethesda titers was lower showed a better response [18].

#### Immunoadsorption / plasmapheresis

This may be a useful modality in patients with very high level of inhibitors titer who failed to respond to the bypassing agents. For the achievement of hemostasis after pheresis or immunoadsorption, FVIII should be replaced in these patients [38].

Rituximab: In acquired hemophilia patients, Rituximab has arisen as a golden agent to eradicate the inhibitors [8]. 90–100% complete remission has been achieved of the published data available. The therapeutic dose of 375 mg/m2 per week should be used for one month. The result can be seen in the 1st two weeks of the treatment and the general opinion is that it should be considered for cases who are unable tolerate the standard immunosuppressant drugs or show resistance to first line treatment. Rituximab in combination with prednisolone is also proposed by some groups as first line of therapy in the patients with inhibitor titer from 5 to 30 while for the patients with a titer greater than 30 it should be used in addition to prednisolone and cyclophosphamide [46].

## Conclusion

Here we reported two cases of SAHA which were diagnosed after multiple surgeries, the SAHA was recognized by its general characteristics of having continues bleeding, high aPTT, low factor VIII activity, high level of factor VIII inhibitors and lack of previous personal and family bleeding history. The mortality rate in SAHA is high due to the deficient awareness or late recognition of the disease. Therefore, it requires prompt diagnosis on the basis of SAHA’s specific diagnostic tests and growing awareness of physicians of various specialties. In addition, a two-pronged approach including resumption of hemostasis and eradication of factor VIII inhibitors are required for excellent treatment of SAHA.

## Data Availability

The datasets used during the current study are available from the corresponding author on reasonable request.

## Abbreviations

AHA: Acquired Hemophilia A
SAHA: Surgery associated acquired haemophilia A
DSA: Digital subtraction angiography
aPTT: Activated partial thromboplastin time
BU: Bethesda units
rpFVIII: Recombinant porcine factor VIII
rFVIIa: Recombinant factor VIIa
APCC: Activated prothrombin complex concentrates
DDAVP: 1-deamino-8-D-arginine vasopressin
FEIBA: Factor eight inhibitor bypass activity
IVIG: Intravenous immunoglobulin
INR: International normalized ratio
